# Pro-Inflammatory Signalling PRRopels Cisplatin-Induced Toxicity

**DOI:** 10.3390/ijms23137227

**Published:** 2022-06-29

**Authors:** Ivan K. Domingo, Asna Latif, Amit P. Bhavsar

**Affiliations:** Department of Medical Microbiology and Immunology, University of Alberta, Edmonton, AB T6G 2E1, Canada; ivankris@ualberta.ca (I.K.D.); asna@ualberta.ca (A.L.)

**Keywords:** cisplatin, toxicity, inflammation, pro-inflammatory, signalling, cytokines, chemokines, combinatorial therapy

## Abstract

Cisplatin is a platinum-based chemotherapeutic that has long since been effective against a variety of solid-cancers, substantially improving the five-year survival rates for cancer patients. Its use has also historically been limited by its adverse drug reactions, or cisplatin-induced toxicities (CITs). Of these reactions, cisplatin-induced nephrotoxicity (CIN), cisplatin-induced peripheral neuropathy (CIPN), and cisplatin-induced ototoxicity (CIO) are the three most common of several CITs recognised thus far. While the anti-cancer activity of cisplatin is well understood, the mechanisms driving its toxicities have only begun to be defined. Most of the literature pertains to damage caused by oxidative stress that occurs downstream of cisplatin treatment, but recent evidence suggests that the instigator of CIT development is inflammation. Cisplatin has been shown to induce pro-inflammatory signalling in CIN, CIPN, and CIO, all of which are associated with persisting markers of inflammation, particularly from the innate immune system. This review covered the hallmarks of inflammation common and distinct between different CITs, the role of innate immune components in development of CITs, as well as current treatments targeting pro-inflammatory signalling pathways to conserve the use of cisplatin in chemotherapy and improve long-term health outcomes of cancer patients.

## 1. Introduction

**Figure 1:**
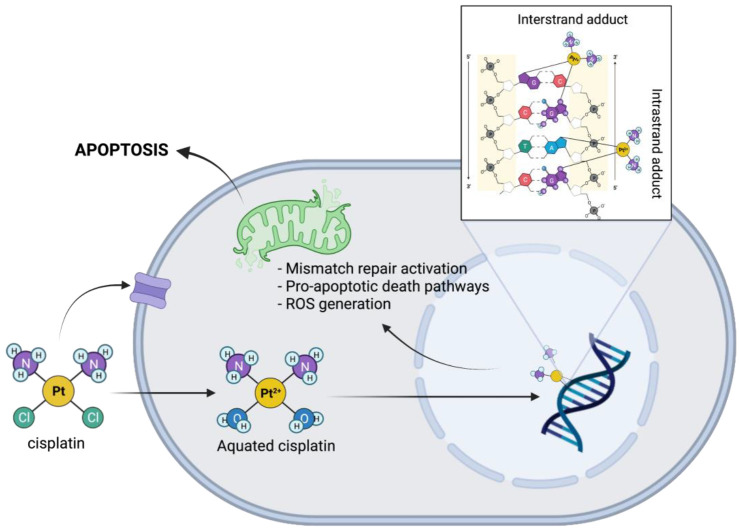
Mechanism of action of cisplatin in tumour cells. Cisplatin enters the cell either passively or through a transporter, where two water molecules replace the chloride groups. As an electrophile, cisplatin is attracted to the nitrogen on purine bases in the DNA, where it forms inter or intra-strand crosslinks, interrupting DNA repair and replication processes. Downstream this leads to oxidative stress and activation of apoptotic signalling pathways.

*Cis*-diaminedichloroplatinum (II), or cisplatin, is a powerful chemotherapeutic agent that has been in use for decades to treat a multitude of cancers alone or in combination therapies. Its ability to inhibit cellular division was first discovered by Dr. Burnett Rosenburg in 1965—120 years after it was first synthesised—it was licensed for medical use in chemotherapy shortly after, in 1970 [1,2,3]. As the first platinum-based antineoplastic drug approved by the FDA, it has since then pioneered research and development into countless other transition-metal based chemotherapies [4]. Specifically, the anti-tumoural properties of cisplatin were linked to its ability to restrict cell division through its intercalation into the DNA of reproducing cells [5,6,7,8]. Cisplatin is aquated when it enters the cell and attacks the purine bases in the tumoural DNA, with particular affinity for the N^7^ of guanine. The formation of intra-strand adducts by covalently binding adjacent guanine or adenosine bases was found to be strongly associated with its cytotoxic effects, though inter-strand adducts are also formed with less frequency, as shown in Figure 1. This distorts the DNA structure and recruits mismatch repair machinery to the nucleus, but ultimately an inability for cells to repair their DNA induces oxidative stress, cell cycle arrest, and initiation of pro-apoptotic pathways [5,6,7]. This mechanism of action allows it to target tumour cells with particular preference due to the accessibility of replicating DNA and high basal levels of reactive oxygen species. The simplicity and efficacy of its anti-cancer activity has allowed cisplatin to become an indispensable drug in cancer therapy, contributing to improved survival rates in solid-state cancers. Since then, more and more attention has shifted to the adverse drug reactions caused by cisplatin chemotherapy. Despite its efficacy in treating solid-state cancers ranging from the head-and-neck [9,10], to ovarian [11,12], and testicular [13,14], and several others [15,16], especially childhood-related cancers [17,18,19,20,21,22,23], the toxicity of cisplatin has put serious limitations on its clinical use as well as the long-term health outcomes and quality of life of cancer survivors. The adverse drug reactions caused by cisplatin and its toxicity profile have led to the development of several cisplatin-induced toxicities (CITs) [24,25,26], which appear to target specific parts of the body such as the kidneys, liver, neurons, and inner ear. This is due to the preferential accumulation of cisplatin in these regions following repeated intravenous administration during treatment [15,27,28]. As such, some of the most commonly reported CITs include cisplatin-induced nephrotoxicity (CIN) [27,29,30], cisplatin-induced hepatotoxicity (CIH) [31,32], cisplatin-induced peripheral-neurotoxicity (CIPN) [33,34,35], and cisplatin-induced ototoxicity (CIO) [36,37,38].

Over the years, several methods have been developed to alleviate the effects of cisplatin, though none have proven to be effective as a generic countermeasure against all forms of CIT due to the diverse nature of its activity and differences in responses to cisplatin at distinct target sites. 

**Figure 2:**
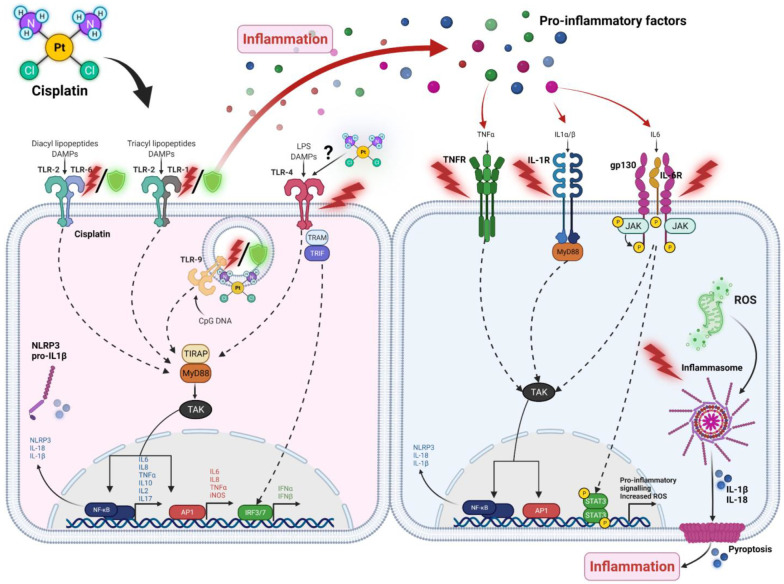
Pattern recognition receptors and pro-inflammatory signalling pathways involved in cisplatin-induced toxicities. Pattern Recognition Receptors interact with a specific array of PAMPs and DAMPs to mediate signals that induce pro-inflammatory signalling. Most of these pathways share downstream signalling that converge on NF-κB, AP-1, or IRF3, which regulate expression of pro-inflammatory signalling molecules such as cytokines and chemokines to influence inflammation in surrounding cells. These molecules bind receptors like TNF-R and IL6-R in other cells that mediate similar signalling pathways. This localised inflammation is exacerbated by cisplatin treatment and is involved in various CITs through mechanisms that remain to be elucidated.

**Figure 3:**
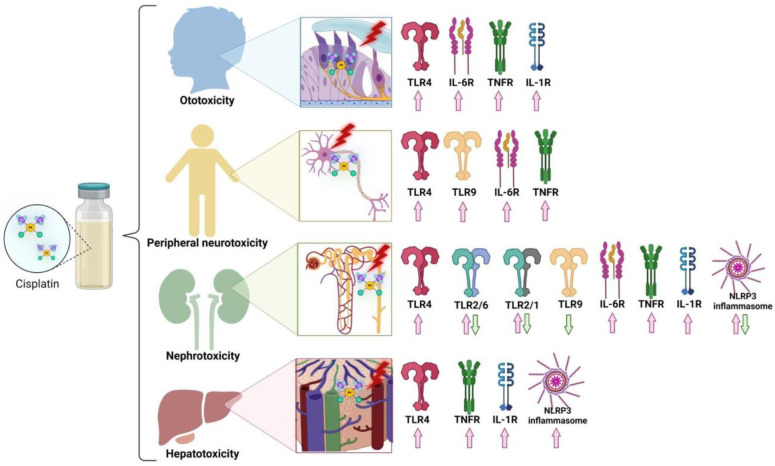
Summary of reported innate immune receptors involved in cisplatin-induced ototoxicity, peripheral neurotoxicity, nephrotoxicity, or hepatotoxicity. The depicted innate immune receptors are involved in either protecting (green arrow) or exacerbating (red arrow) inflammation in response to systemically delivered cisplatin, and a few key receptors have been reported in both cases.

This has led to two primary branches of investigation: one which seeks to develop drugs related to cisplatin which are intrinsically less toxic (such as carboplatin and oxaliplatin), and one which seeks to identify the underlying mechanisms driving the development of CITs so that they may be specifically targeted and inhibited, ideally without affecting treatment efficacy; see Figure 2 for a summary of specific signalling pathways activated by cisplatin. The mechanism-based studies seek to preserve the use of cisplatin in chemotherapy and potentiate its use in clinic, but most research seeking to prevent CITs has been focused on the nature and impact of reactive oxygen and nitrogen species, which are known to be the downstream damage-inducing agents in CITs; however, this branch of research does not focus on identifying the actual instigators of cisplatin-induced damage and subsequent oxidative stress. Instead, there has been rising interest in another phenomenon that has appeared to be common amongst nearly all forms of CIT: inflammation. Hallmarks of inflammation have been used to gauge the severity of CITs since they were first characterised, and the role of cisplatin-induced inflammation as a direct initiator of the damage associated with CIT development has grown to be an especially hot topic of research; see Figure 3 for a summary of the inflammatory mechanisms contributing to each major CIT. Increasingly, natural and synthetic anti-inflammatory compounds with the potential to significantly ameliorate CITs have become more prominent and promising.

In this review, we highlighted the immunological traits of cisplatin-induced toxicities and covered the importance of pro-inflammatory signalling systems in their development, especially through the innate immune system. We also discussed the prospects of anti-inflammatory agents as protective combinatorial therapies centred on immunomodulation to curtail CITs and preserve and potentiate the use of cisplatin in cancer therapy. 

### Inflammation and Pro-Inflammatory Signalling

Immune responses can be classified as either ‘innate’ or ‘adaptive’. Adaptive immune responses are activated in response to persistent infection and/or damage; they are tailored to facilitate responses to specific forms of antigens and are carried out by adaptive immune cells, such as helper and cytotoxic T-cells, and antibody-producing B-cells. Innate immune responses are, conversely, immediate, non-antigen-specific, responses that can be triggered by the vast majority of cells and mediated by myeloid phagocytic cells. These include, but are not limited to, neutrophils, macrophages, and monocytes [39,40].

The process of inflammation is predominantly handled by the systems associated with the innate immune system which rely on ‘input signals’ generated by pattern-recognition receptors (PRRs), as well as cytokines and chemokines and their respective unique receptors. 

PRRs bind common pathogen-associated molecular patterns (PAMPs) and initiate downstream signalling cascades that culminate in the expression of genes required to recruit dedicated immune cells and kickstart inflammation. Often, these PRRs also double as damage-recognition receptors, in that they can also bind and trigger responses to host cellular damage-associated molecular patterns (DAMPs), such as extracellular genomic DNA and mitochondrial DNA (mtDNA), and heat shock proteins. PRRs can be classified into four main categories, or families: (1) Toll-Like Receptors (TLRs), (2) Nucleotide-Binding Oligomerisation Domain (NOD)-Like Receptors (NLRs), (3) Retinoic-Acid-Inducible-Gene (RIG)-Like Receptors (RLRs), and C-Type Lectin Receptors (CLRs) [41].

TLRs localise to the cell surface or intracellular compartments and are thus prime to detect extracellular or vesicular signs of pathogens, such as bacterial lipopolysaccharides (LPS) [42]. They are also responsible for the detection of most DAMPs when paired with specific accessory proteins or coreceptors [43]. Certain TLRs have also been implicated in the development of unique hypersensitivity/allergic reactions [44,45,46,47]. NLRs, conversely, are responsible for the recognition of intracellular signifiers of infection, such as bacterial peptidoglycan components (iE-DAP), bacterial toxins, and viral nucleic acid structures [48,49]. Upon exposure to any of these potential agonists, NLRs trigger downstream signals that reflect that of TLRs but also facilitate the creation of complexes known as inflammasomes that can additionally modulate/control cell survival/death systems [50,51,52]. Like NLRs, RLRs are also responsible for detecting intracellular pathogens, but are equipped to specifically bind common genetic elements of both viral and intracellular bacterial pathogens [53,54,55]. CLRs, on the other hand, are more akin to TLRs, binding pathogen-associated carbohydrates from the extracellular space to cause pro-inflammatory gene expression changes identical to that of most other PRRs. Unlike TLRs, however, CLRs have a more direct role in arbitrating the transition from innate to adaptive immune responses by using captured antigens for presentation and provision to dendritic cells that can explicitly activate adaptive immune cells [56,57]. 

The Toll-like Receptors each bind a specific array of PAMPs and DAMPs and, through several unique and shared downstream signalling systems, activate three key transcription factors, nuclear factor kappa B (NF-κB), activator protein-1 (AP-1), and interferon regulatory factor 3, (IRF3), responsible for enabling the expression and secretion of soluble pro-inflammatory signalling molecules such as cytokines and chemokines.

Most, if not all forms of PRR, conclude in the activation of at least one of three key gene transcription factors: (1) AP1, (2) NF-κB, and (3) IRFs. It is through these three transcription factors that the state of inflammation can be modulated [58]. AP1 consists of homo- or hetero-dimeric complexes which consist of four types of DNA-binding proteins: Jun-family proteins, Fos-family proteins, activating transcription factors/cAMP response element binding protein (ATF/CREB)-family proteins, and musculoaponeurotic fibrosarcoma (MAF) family proteins. Different combinations of these subunits, in the context of different cells and conditions, can lead to distinct gene expression profiles, including the upregulation of pro-inflammatory cytokines and chemokines [59,60,61]. NF-κB can similarly mediate the upregulation of pro-inflammatory cytokines and chemokines, and can be considered the primary regulator of inflammation given that it can play a pivotal role in micromanaging both innate and adaptive immune responses. That said, NF-κB is also the ‘master regulator’ for countless other homeostatic genes, allowing it to also alter cell cycle progression in conjunction with inflammasomes and other inflammatory factors, activate dedicated immune cells to manipulate their maturation and differentiation processes, and influence the expression of tertiary features with additional roles in inflammation, such as adhesion molecules [62,63,64]. Juxtaposed to the prior two transcription factors, IRF3 contributes to inflammation by promoting the secretion of a unique class of cytokines, called Type I Interferons. Unlike AP1 and NF-κB, which are targets shared between almost all PRR downstream signalling mechanisms, IRF3 is under the purview of only a specific subset of PRRs—namely, TLR members, TLR3 and TLR4, as well as RLRs. Its activity is also under the control of a limited set of non-PRR, DAMP-specific, pro-inflammatory complexes such as cGAS-STING [65,66]. 

Cytokines and chemokines, unlike PRRs that centre on ‘paracrine’ interactions, can operate as autocrine and endocrine means of cellular communication, and can direct the development of inflammation through self-reinforcing signals and long-distance signalling. Both cytokines and chemokines are small proteins that can vary wildly in function and can either be pro-inflammatory or anti-inflammatory. Typically, cytokines incite their own set of downstream signalling events to alter gene expression while chemokines mainly operate as chemoattractants—recruiting different subsets of immune cells. 

Pro-inflammatory cytokines and chemokines in particular are produced and released as a result of PRR signalling cascades—each PRR family is responsible for the production and release of specific cytokines and chemokines. The most common pro-inflammatory cytokines are the interleukins (IL), IL-1β and IL-6 especially, as well as Tumour Necrosis Factor-α (TNF-α). IL-1β has been identified as a critical factor in the development of fevers and the pain responses associated with inflammation. IL-6 has been shown to activate acute phase responses (APRs)—a set of changes to serum protein concentrations—intended to render adjacent areas inhospitable to any pathogens present. TNF-α, on the other hand, helps protect against intracellular threats by making host cells inhospitable environments and contributing to pro-apoptotic signalling systems. 

The most common chemokines in comparison tend to be CXC-motif chemokine 2/keratinocyte-derived chemokine (CXCL2/KC) and CXC-motif chemokine 8/interleukin-8 (CXCL8/IL-8), which control the recruitment of neutrophils as T-cells to sites of infection/inflammation [67,68]. ‘Positive’ acute-phase proteins are proteins that are increased to promote inflammation, and include, but are not limited to complement factors, degradative enzymes, and iron chelators, all of which can also hurt the host with prolonged exposure [69,70].

## 2. Pattern Recognition Receptors (PRRs) in Cisplatin-Induced Toxicities

### 2.1. Toll-Like Receptors (TLRs)

Toll-Like Receptors (TLRs) have been one of several features of immunology heavily studied in association with cisplatin-induced toxicities. Of the ten different members of the TLR family, two appear to have a major influence on the progression of CITs and the damage they can ultimately do: TLR4 and TLR2 (Figure 2). 

TLR4 presents on the cell membrane as a homo-dimer and is special in that it can transduce signalling through two intracellular pathways associated with TLRs: Myeloid differentiation primary response 88 (MyD88) dependent and Myd88 independent pathways [71]. MyD88 is an adaptor molecule that allows for signal convergence following TLR stimulation and while other TLRs utilise one or the other pathway, TLR4 can use both, allowing for significant crossover and potentiation of signalling. It is well known for being stimulated by a bacterial PAMP called lipopolysaccharide, a component of gram-negative bacterial cell membranes, as well as some DAMPs with the help of various co-receptors. This allows it to detect bacterial infection as well as surrounding cell damage, and is necessary for stimulating a localised immune response and maturation of adaptive immune cells. It has also been shown to mediate allergic hypersensitivity reactions to transition metals, such as nickel, cobalt, and palladium [44,45,46,47]. Importantly, TLR4 is associated with co-receptors, myeloid differentiation factor 2 (MD2), and cluster of differentiation 14 (CD14), which are necessary for signal transduction in response to lipopolysaccharide [42]. In the context of CIO, TLR4 appears to play an important role in exacerbating inflammation in the inner ear. Inhibition of TLR4 chemically via small molecule inhibitors or through gene silencing has been shown to confer protection in the context of the three most prevalent CITs (CIN, CIPN and CIO), as well as rarer cases, such as cisplatin-induced hepatotoxicity (CIH) [72]. 

Though the exact mechanism(s) driving the relationship between CITs and TLR4 have yet to be completely elucidated, several aspects have been typified, and a number of theories proposed. For example, the activation of TLR4 mitogen-activated protein kinase (MAPK) pathway-associated proteins, c-Jun N-terminal kinase (JNK) and p38, appear to correspond with toxicity in CIN and inversely correlates with increased cell viability in TLR4-deficient model organisms [72]. In studies, bone marrow chimeric mice have been used to showcase the importance of localised cell TLR4 proteins compared to responding immune cell TLR4s [72]. Transforming growth factor-β-activated kinase 1 (TAK1), a key component of the TLR4 MyD88-dependent signalling pathway, has also shown promise as a potentially valuable target for protecting against CIN. The inhibition of TAK1 leads to reduced activation of the extracellular signal-regulated kinase (ERK) and p38 MAPK signalling pathways through TLR4—leading to a reduction in tubular damage in the kidneys [73,74].

In cases of CIO, compounds that block MAPK activation, akin to the calcium channel antagonist Flunarizine, have been shown to prevent the progression of downstream TLR4 functions as well [75]. Directed inhibition of ERK caused a significant reduction to typical levels of NF-κB activation and pro-inflammatory cytokine secretion—sufficient to increase cell viability [75]. It has also been demonstrated that LPS-mediated TLR4 activation has a synergistic effect on CIO and that cisplatin induces the upregulation of TLR4, increasing the extent of toxicity and subsequent hearing loss [76]. This implies that TLR4-mediated induction of inflammation may be a separately looping but nevertheless contributing factor to CITs. This same principle has been found to apply to CIN and endotoxin insult and septic shock [77].

On the other hand, numerous studies exist indicating that cisplatin-induced pro-inflammatory responses can develop even in presumptively sterile conditions and in an MD2-independent manner. In 2021, Babolmorad et al. demonstrated that cisplatin-induced pro-inflammatory cytokine secretion could be induced in human embryonic kidney cells that express TLR4 but do not express MD2 [78]. The inhibition of TLR4 activation through commercially available chemical inhibitors like TAK242, and the prevention of TLR4 expression through siRNA and clustered regularly interspaced short palindromic repeats (CRISPR), all proved sufficient to reduce CIT-associated proinflammatory responses in the same system [78]. Moreover, it was also demonstrated that TLR4 may specifically interact with the platinum component of cisplatin to mediate inflammation as it has been shown to do so for metal allergens, suggesting that potentially direct interactions between cisplatin and TLR4 could be stimulating CIO; however further evidence is required to confirm this. In 2015, TLR4-deficient mice and MyD88/TRIF-deficient mice obtained significant to near-absolute protection from CIPN signified by mechanical allodynia (pain sensitivity) [79,80]. Transcriptomic analyses have identified seven genes that appear to dictate CIPN severity, almost all of which are linked to immunity, including *TLR4* [81]. Ibudilast and isomers of the opioid antagonists, Naltrexone and Naloxone, have been shown to inhibit TLR4 activity and have all been shown to reduce chemotherapy-induced peripheral neuropathies similar to CIPN as well [82,83,84]. The opioid antagonists are believed to operate by co-opting MD2 to bind and block TLR4 [85]. Recent data suggests that Ibudilast can directly interact with and inhibit interleukin-1 receptor-associated kinase 1 (IRAK1) to inhibit the MyD88-dependent signalling pathway of TLR4—among several of its other indirectly anti-inflammatory modes of action as a phosphodiesterase inhibitor [86]. 

In contrast to TLR4, TLR2 has a protective role against CITs. TLR2 exists primarily on cell surface membranes such as TLR4, but is expressed exclusively and pre-emptively as part of heterodimers with either TLR1 or TLR6. TLR2 facilitates the recognition of and innate immune responses to a variety of multi-acylated lipopeptides as opposed to lipopolysaccharides. Multi-acylated lipopeptides, specifically di- and tri-acylated lipopeptides, can be found in bacteria, viruses, fungi, and even parasites, making TLR2 an extremely versatile PRR. TLR2 can nevertheless also mediate responses to typical DAMPs, such as heat shock proteins and high mobility group box 1 protein (HMGB1). 

Like other TLRs, TLR2 depends on particular coreceptors to bolster the recognition of particular PAMPs and DAMPs. Along with CD14, CD36 also assists with TLR2 activation in the vast majority of responses. Most TLRs are also known to have one downstream signalling pathway in common—the MyD88-dependent downstream signalling pathway, and TLR2 is no exception. Upon binding a ligand, TLR2 initiates the MyD88-dependent downstream signalling cascade but differentiates itself from *TLR4* in that it must undergo internalisation first to do so, and does not signal through the MyD88-independent signalling pathway [42,87]. At least two independent studies have shown that TLR2 activation can provide a considerable degree of protection from CITs. Removal of TLR2-enhanced CIN specifically, coinciding with a reduction in the markers of autophagy, and a shift to immunosuppressive cytokine regimens and adaptive immune responses by resident dendritic cells [88,89,90]. Renal stem cell recovery systems, which can limit cisplatin-induced acute kidney injuries, appear to be at least partly dependent on TLR2 activation as well [91]. Some studies have shown that depletion of TLR2 is associated with protection and subsequent reductions in pro-inflammatory IL-17A in CIN, and especially when there is simultaneous inhibition of TLR4 expression or activity. However, there are also studies that connote to the opposite [92,93]. Reports on its role in CITs are limited though and it poses an intriguing area for further investigation, particularly since it is not clear which heterodimer is more involved in this process—TLR2/TLR1 or TLR2/TLR6. 

Little more is known about the connection between TLRs and CITs beyond that. The TLR4 and TLR2 agonists present or released following cisplatin treatment and responsible for dictating the course of cisplatin-induced toxicities have remained unknown. TLR9 has also recently drawn attention as new reports indicate that it too may play a role in CITs, albeit differently. TLR9, another TLR PRR, is canonically geared towards the recognition of DNA structures associated with pathogens (unmethylated CpG-DNA motifs), but it has also been shown to recognise DNA-related DAMPs and bacterial by-products and components [42,94]. As such, TLR9 typically exist in distinct types of endosomes that dictate the weighting of their signalling cascades towards either pro-inflammatory IRF-dependent interferons or the typical NF-κB-related repertoire of cytokines and chemokines [42]. In CIN, it surprisingly limits neutrophil invasion and mediates the recruitment of protective adaptive immune cells, T-Regulatory-Cells (T_Regs_) [95]. Cases of chemotherapeutic peripheral neuropathy not necessarily limited to just cisplatin alternatively suggest that TLR9 may instead exacerbate CIPN through the recruitment and activation of pro-inflammatory macrophages [96].

Regardless, while the link between specific TLRs and CITs appears to be robust, it is not absolute. Neither the inhibition of TLR4, nor the expression of TLR2, can guarantee complete protection from cisplatin-induced toxicities, suggesting that other factors are also at play, some of which may be immunological in nature, but not entirely dependent on TLRs alone. 

### 2.2. NOD-Like Receptors (NLRs) and Inflammasomes

Similar to TLRs, nucleotide-binding and oligomerisation domain (NOD)-like receptors (NLRs) are also a type of pattern-recognition receptor family. Unlike TLRs, they operate exclusively in the cytosol and detect various pathogenic and damage signals as well as homeostatic disruptions in the cell to stimulate a signalling cascade and facilitate activation of the innate and adaptive immune systems [97,98]. The NLR family is further divided into sub-families, each of which are defined by their distinct N-terminal domains, leading to various overlapping signalling pathways that either activate transcriptional activators or lead to the assembly of inflammasomes, which are large protein complexes in the cytosol that catalyse apoptotic effector functions. In this review, we do not detail the different kinds of NLRs and their signal transduction but instead focus on NLRs that appear to be most closely associated with CIT development (Figure 2). For an extensive discussion on NLRs and their involvement in human disease, please see the excellent review by Zhong et al. (2013) [97]. 

Of the various types of NLRs in mammalian cells, the one that appears to be the most involved in CIT is the inflammasome-forming NLRP3. While many PRRs are specific to a certain subset of activating signals, NLRP3 signalling is induced by a wide array of PAMPs and DAMPs [99]. The activation of the NLRP3 inflammasome is complex and involves many mediators that lead to increased cell stress; however, in a simplified model, the sensing of cell stress by NLRP3 is typically preceded by a two-step process [97]. First, there must be an upregulation of NLRP3 and other inflammasome factors such as caspase 1, which carries out the effector functions of the NLRP3 inflammasome, and pro-IL-1β, which is the inactive form of a pro-inflammatory cytokine that is cleaved by caspase 1. This first step is called the priming step and occurs through activation of other PRRs and receptors in response to their respective PAMPs and DAMPs. Interestingly, some of these priming responses overlap with inflammatory signalling pathways we have seen to be involved in CIT development, including TLR4-LPS interactions or TNFα and IL-1β stimulation [97,100]. The priming leads to NFκB-mediated transcription of inflammasome components so that an inactive form of NLRP3 in the cytosol is prepared to respond to cell stress. The second step is the activation step of the NLRP3 inflammasome, and is mediated by various stimuli that disturb cell homeostasis such as changes in K^+^ and Cl^−^ efflux or lysosomal disruption, to name a few. The DAMPs or PAMPs involved in stimulating these cellular disturbances are quite diverse and are not known to interact directly with NLRP3 itself; rather, NLRP3 senses these changes through unknown mechanisms and becomes “activated”, leading to its oligomerization and initiation of inflammasome formation. The pyrin domain at its N-terminus allows for interaction with apoptosis-associated speck-like protein containing a CARD (caspase activation and recruitment domain) (ASC), an adaptor that can then interact with caspase 1 and complete the assembly of the inflammasome [101,102]. Ultimately, the inflammasome allows for caspase 1-mediated cleavage of the inactive form of IL-1β and IL-18 to active forms, as well as cleavage and release of the pore-forming gasdermin D, which leads to localised cytokine-mediated inflammation or cell death through pyroptosis, respectively. 

The role of NLRP3 in CIT is most commonly reported in the kidneys, specifically owing to the similarity between the inflammatory profile of NLRP3-mediated diseases, ischemic (non-chemically induced) acute-kidney injury (AKI) and cisplatin-induced AKI [102,103,104]. This fostered interest in the role of NLRP3 in cisplatin-induced nephrotoxicity.

Cisplatin-induced renal injury is generally associated with an increase in NLRP3 inflammasome components, but there are conflicting reports about its significance. In a study investigating cisplatin-induced renal dysfunction, younger male C57BL/6 mice were found to have increased IL-1β, IL-18, ASC, caspase 1, as well as NLRP3 in the kidneys 3 days following treatment [105]. This study specifically looked at the role of a receptor, purinergic receptor P2X7 (P2X7R), in exacerbating CIN through NLRP3 activation. This receptor has been reported to induce NLRP3 inflammasome activation by responding to extracellular ATP, a DAMP, and inducing pore-formation and K^+^ efflux, acting as a component of the second activation step of NLRP3 stimulation [106,107]. Chemical inhibition of this receptor protected mice from renal dysfunction and injury, reduced levels of inflammasome components and pro-inflammatory cytokines, and protected them from oxidative stress and apoptosis. Thus, reduction of inflammasome components following cisplatin treatment was associated with protection against CIN. Similarly, several other reports found an increase in NLRP3 inflammasome activity following cisplatin treatment that was associated with kidney injury [108,109,110,111,112,113]. Specifically, they found increases in inflammasome components, pro-inflammatory cytokines like IL-1β, IL-18, TNF-α, or even increased pyroptotic activity. Stimulation of pyroptosis in CIN occurred through increased levels of Gasdermin D, a protein involved in mediating pyroptosis that is also a substrate of the NLRP3 inflammasome [111]. Cisplatin-induced NF-κB activity increased NLRP3 inflammasome components as well as pyroptotic activity in mouse kidneys, all of which could be ameliorated by vitamin D-induced downregulation of NF-κB. Many of these studies assess processes that occur upstream of NLRP3 activation such as cisplatin-induced mitochondrial dysfunction or NF-κB upregulation, which speaks to its role as a downstream effector of cisplatin-induced inflammation, but not necessarily a causative agent of CIN. The most direct evidence for NLRP3 involvement in CIN comes from NLRP3-specific inhibitor experiments that showed protection against CIN. Cisplatin-induced kidney injury was mitigated by chemical inhibition of NLRP3 with MCC950, demonstrating the role of the NLRP3 inflammasome in exacerbating CIN directly [114]. 

In contrast to this, one study found that NLRP3 knockout mice were not protected from cisplatin-induced AKI and there was little to no change in the pro-inflammatory cytokine profile in cisplatin-treated wild type versus knockout mice [115]. While they did find that caspase 1 knockout mice were protected against cisplatin-induced apoptosis and renal failure [116], follow up studies provided weak evidence that inhibition of NLRP3 activity protected against cisplatin-induced AKI [115]. Kim et al. (2013) also found an increase in ASC and caspase 1 in older male C57BL/6 mice after cisplatin treatment, indicating increased NLRP3 inflammasome activity; however, there was no significant increase in the NLRP3 protein itself in the mouse kidney [115]. 

Similarly, Kim et al. (2013) found no increase in IL-1β in-vivo and mouse macrophages did not show a cisplatin-induced increase in NLRP3 in-vitro, thereby discounting the idea that NLRP3 mediated damage could be coming from invading innate immune cells [115]. While the authors did find that the NLRP3 inflammasome appeared to be involved in ischemic AKI, they concluded that it has no role in cisplatin-induced AKI. Interestingly, NLRP1 (a different NLRP-inflammasome) and its inflammasome effector caspase, caspase 5, were shown to be increased in mouse kidneys following cisplatin treatment. This suggests a role for NLRP1 in cisplatin-induced AKI. Even more intriguing was their finding that NLRP3 knockout decreased NLRP1 levels following cisplatin treatment, implicating a dependence of NLRP1 on NLRP3 in CIN. Moreover, NLRP3 inflammasome activity is associated with various kinds of kidney injury [117,118,119]. While cisplatin may induce its activity, the role of the NLRP3 inflammasome in CIN may work in concert with the injury cisplatin causes in the kidney through other mechanisms.

Due to its reliance on several exogenous signals for activation, the NLRP3 inflammasome is in a position where its involvement in CITs is closely tied to other inflammatory pathways, including other PRRs. Namely, TLR4 stimulation leads to an NF-κB-mediated increase in NLRP3 in response to cisplatin treatment in kidneys [108]. Cisplatin induces upregulation of all of these factors, and stimulation of TLR4 allows priming of NLRP3 components that are later activated by cisplatin-induced stress. A proton-pump inhibitor called omeprazole, which should only affect the NLRP3 inflammasome activation portion of this signalling axis, was effective in ameliorating CIN and decreasing TLR4/NF-κB/NLRP3 levels. In this way, signal convergence from other PRRs like TLR4 may facilitate NLRP3-mediated CIN. 

Along with CIN, cisplatin-induced liver toxicity is also associated with NLRP3 inflammasome activity, though this is far less studied. Cisplatin-treated rat liver has increased NLRP3 protein, IL-1β, and caspase 1 that is correlated with increased oxidative stress, inflammation, and liver injury [112,120]. These effects were reversed by compounds that are suggested to inhibit factors such as NF-κB and MAPK, both of which work upstream of NLRP3 and, in the case of NF-κB, are involved in priming of NLRP3 inflammasome components. While this demonstrates its involvement, the direct role of NLRP3 in exacerbating inflammation in CIH remains to be seen with specific inhibition of NLRP3 in hepatocytes or gene silencing. 

While the abundance of evidence indicating its significance in CIN and CIH imply NLRP3 to be a fascinating target for protective therapy, suppression of NLRP3 has also been associated with increased tumour resistance to cisplatin [121]. Cisplatin-resistant tumour cells in non-small lung cancers had downregulated NLRP3 and upon upregulation of NLRP3, tumour cells were once again sensitised to cisplatin treatment. This means that, while it may very well be involved in exacerbation of CITs, it may not be an ideal target for protection during cancer therapy as it potentially compromises the efficacy of cisplatin’s anti-tumoural activity.

## 3. Pro-Inflammatory Messengers (Cytokines and Chemokines)

Resulting from the downstream signalling of PRRs and the consequent activation of gene transcription factors such as NF-κB are the pro-inflammatory cytokines and chemokines. Pro-inflammatory cytokines and chemokines, such as IL-1β, IL-6, IL-8, and TNF-α, essentially serve as easily quantifiable indicators of cisplatin-induced toxicities since they are expressed directly proportional to the severity of conditions (Figure 2). In all of the prior publications mentioned, increases in toxicity (such as with TLR2 inhibition) corresponded with increases in IL-6, IL-1β, and TNF-α by at least 20–50%, while protection from toxicity corresponded with reductions in secretion by up to 50–80%. This relationship is also directly related to the dose of cisplatin used and the exposure time.

All in all, the relationship between cytokine secretion and cisplatin toxicity does not appear to be linear either; pro-inflammatory cytokines appear to actively contribute to the pathology of prolonged cisplatin exposure in a positive-feedback loop. So et al. (2007) demonstrated that the provision of recombinant exogenous pro-inflammatory cytokines associated with CITs could elicit cell viability loss capable of accounting for up to 20% of the cell death associated with CIO [122]. More importantly, the group also showed that the targeted depletion of pro-inflammatory cytokines through blocking antibodies could sufficiently mitigate the death caused by cisplatin treatment, the most effective of which being anti-TNF-α [122]. In 2007, Zhang et al. used chimeric mouse models to demonstrate that the expression of TNF-α by immune cells residing in the kidney was critical for CIN development as well [123]. That said, the impact of IL-6 expression remains contentious in CIN, as Faubel et al. indicated that IL-6 deficiencies, alone, provide little to no respite from CIN [124]. Corroborating evidence came from Kim et al. (2011) who discovered that STAT6−/− mice produced far less of the characteristic three pro-inflammatory cytokines and were accordingly protected from CIO [125]. Like their predecessors, they reported that anti-IL-6 and anti-TNF-α provided the greatest resistance to cisplatin cytotoxicity. It may also be important to note that pro-inflammatory cytokines, like TNF-α, have the capacity to trigger conditions associated with CIPN—not just CIO—as well. Both the natural upregulation of pro-inflammatory cytokines by injured Schwann cells, and the injection of exogenous pro-inflammatory cytokines like TNF-α, have the potential to provoke mechanical allodynia and thermal hyperalgesia—common manifestations of CIPN [126,127]. In-vitro, the provision of TNF-α also confers a permanent degree of hyper-responsiveness that would presumably lead to pain, and hypersensitivity to subsequent immune factors [126,127]. This is likely due to the pleiotropic effector functions of both the IL-6 and TNF-α and their receptors. IL-6 receptors (IL6R/CD126/gp80) can exist as either membrane-bound or cytosolic receptors, though they typically exist in their soluble “trans-signalling” form in non-immune cells [128,129]. Soluble IL6R is primarily responsible for pro-inflammatory induction through the activation of Janus kinase/signal transducer and activator of transcription 3 (JAK/STAT3) and Src-homology region 2 (SH2)-containing protein tyrosine phosphatase 2 (SHP2)/MAPK pathways which exert control over monocytic differentiation, vasculature, immune cell infiltration, and indirectly promote deleterious ROS production [129,130,131]. 

TNF-α receptors (TNFR) also come in two forms, TNFR1 and TNFR2, both of which drastically affect cisplatin toxicities. TNFR1 is considered one the principal drivers of pro-apoptotic signalling as a dedicated ‘Death Receptor’ equipped with a Death Domain that grants access to caspase activation cascades, while TNFR2 is associated with the propagation of inflammation through dedicated immune cell activity [132]. Inhibition of at least one TNFR is enough to confer significant protection from CIN, though the exact importance of each to toxicity remains contentious. Tsuruya et al. (2003) highlighted protection attributed to TNFR1 deficiency, but Ramesh and Reeves (2003) contrastingly reported that TNFR2 deficiency provided a greater degree of resistance to toxicity despite the upregulation of both receptors in murine models of CIN [133,134,135]. Therapeutics designed to mimic TNF receptors, such as etanercept, operate by sequestering available TNF-α from functional membrane forms, and have proven to be sufficient in reducing aspects of CIO in-vivo [136]. 

Note that IL-6, IL-1β, and TNF-α may be the most commonly used indicators of CIT onset but they are historically not the only ones. For example, CIO development in HEI-OC1s has also correlated with the expression of IL-4 and IL-13, which appear to trigger the signalling cascade that leads to the phosphorylation and activation of STAT6. CIO also correlated with an upregulation in IL-5, but it was oddly found irrelevant to pathology [125]. Cisplatin toxicity has also been measured through the proportional upregulation or downregulation of IL-8 in the context of both HEK293 and HeLa cells [78].

Attempts to ascertain the entire scope of cytokine and chemokine profile changes associated with CITs have revealed the relevance of certain effector regulation, including IL-1/IL-1Β, regulated upon activation, normal T-cell expressed, and secreted (RANTES) (CCL5), MCP-1 (CCL2), MIP2 (CXCL2), Macrophage Migration Inhibitory Factor (MIF), IP-10 (CXCL10), KC (murine IL-8), IL-17A, IL-18, IFN-γ, and IL-10 [124,125,134,137,138,139,140,141,142,143,144,145,146,147,148]. However, their role in CITs has only grown in complexity. Cytokines can be extremely multifaceted; the same cytokine can elicit both pro-inflammatory and anti-inflammatory responses depending on circumstance, and this is something that must be contended with in the pursuit of novel therapies. For example, while IL-6 appears to adhere to its canonical designation as a pro-inflammatory cytokine, at least in the context of cisplatin-induced toxicities specifically, IL-4 exerts its influence variably. IL-4 expression is directly related to the severity of CIO, as mentioned previously, but it is inversely related to the severity of CIN and CIPN and is a hallmark of protection paired with IL10 [125,143]. IFN-γ similarly manifests as a pro-inflammatory and toxic effector in CIO (when detectable), but conversely presents far more phenotypically ‘complex’ in models in CIN [125,145,149,150,151]. Ultimately, despite the headway made thus far in the analysis of cytokine and chemokine involvement in CITs, there is clear room for growth in our understanding of the actual underlying mechanisms involved.

## 4. Oxidative Stress and Immunologic Regulation

Cisplatin is well characterised to execute cell damage and death via reactive oxygen species (ROS) and reactive nitrogen species (RNS) [152,153,154]. As such, there was previously an emphasis on employing ROS scavengers and anti-oxidative species such as N-acetyl cysteine, glutathione, or sodium thiosulfate in treatments for CITs, particularly in CIO [155]. As opposed to immune-mediated CIT, the release of ROS occurs downstream in the signalling pathway of cisplatin-induced damage in healthy cells. Because the significance of ROS as the damage-causing agent has been established in CIT, it is only touched on briefly in this review—specifically in the context of cisplatin-induced inflammation. It is becoming more and more apparent that immunologic signalling is the initiator of damage effects under cisplatin treatment, and targeting components upstream of ROS work to block the actual cisplatin interface of this signalling pathway. Many reports highlight the close ties between innate immune stimulation and oxidative stress, with each positively regulating the activity of the other [75,154,156]. Incidentally, immunogenic signalling and oxidative stress appears to be important in potentiating cisplatin’s anti-cancer efficacy as well [157,158]. As such, general targeting of ROS and DAMP signalling in all cells may interfere with cisplatin functionality. While oxidative stress is a widespread effect that plays a role in both CITs as well as cisplatin’s anti-tumour effects, immune receptors appear to be predominantly involved in CIT development. In this way, targeting oxidative stress is becoming a less favorable target to anti-inflammation-based therapy, which could allow selectivity for non-tumour cell protection while not interfering with cisplatin’s anti-cancer activity. 

CIO is associated with a depletion of endogenous anti-oxidant factors and enzymes in the cochlea, as well as an increase in oxidative enzymes such as the NADPH oxidase [154,158], all leading to an increase in oxidative stress under cisplatin treatment. This elevated ROS profile following cochlear injury mirrors effects that are seen following noise-induced injury as well [159]. Consequently, antioxidants protect against both cisplatin-induced and noise-induced hair cell death in the cochlea of the inner ear [154,160]. In keeping with the tightly regulated relationship between ROS generation and immune signalling, increased inflammatory signalling has been found to precede ROS generation in CIO [121], but upregulation of transcription factors involved in anti-oxidation effects like nuclear factor erythroid 2-related factor 2/heme oxygenase-1 (Nrf2/HO-1) can attenuate pro-inflammatory cytokine secretion and resulting CIO [74,161], suggesting a positive feedback mechanism between oxidative stress and inflammation in CITs. Similarly, NADPH oxidases enhance TLR4-mediated inflammation in models of sepsis, where a lack of Nrf2 exacerbates inflammation and ROS generation [156]. It has been suggested that this positive feedback effect in CIO may occur via regulators like STAT1, which enhance both pro-inflammatory cytokine production like TNF-α as well as oxidative species like iNOS [135]. Of course, the role of ROS is not limited to CIO but is also involved in CIN and CIH, where oxidative stress is stimulated by inflammatory mediators, or by cisplatin-induced mitochondrial, endoplasmic reticular, or homeostatic dysfunction [104,112,162,163,164]. 

Cisplatin-induced cell stress and ROS generation facilitate the release of DAMPs which exacerbate inflammation and activation of cell death pathways. For more depth on the role of ROS in CITs, please see a more comprehensive review [153]. For the purposes of this review, our focus is on the role of inflammation as an instigator of CITs as a result of the growing interest in identifying specific mechanisms and targets upstream of cisplatin-induced damage. 

## 5. Anti-Inflammatory Remedies

The prevention and treatment of cisplatin-induced toxicities thus far have been limited. The most common course of action to prevent CIT onset has been to reduce dosage, risking reduced efficacy of chemotherapy. Certain conditions, such as cisplatin-induced nephrotoxicity, do have established standard of care procedures. These often only alleviate symptoms rather than prevent or reverse the damage accumulated throughout the chemotherapeutic process. Some strategies may even pose direct negative effects on the efficacy of cancer treatment. There is thus considerable interest in the use of natural products and the development of pharmaceuticals capable of preventing or ameliorating cisplatin-induced toxicities with extreme specificity. Of the numerous compounds that have been investigated, the most extensively studied have had anti-inflammatory properties as either their primary or secondary mode of action. Other reviews have covered the entire gamut of potential remedies; in the following, there will be a particular focus on the most well-established, and most wide-acting, antioxidant and anti-inflammatory options.

For preclinical, all-natural remedies of CITs, the options are abundant (Table 1). At the same time, there is a strong trend in preclinical research towards repurposing natural compounds for scientific and medical purposes as de novo drug discovery and drug synthesis is both time-consuming and expensive. Notably, there is a discrepancy in trends between preclinical natural remedies (Table 1) and current preclinical pharmacological ‘repurposed’ options (Table 2). Moreover, there is likely a great desire to determine whether ancient forms of medicine hold up to academic scrutiny. Of those chosen and listed, the vast majority do show promise as no-cost or low-cost dietary supplements, derived from naturally occurring, edible plants and/or fauna. Whether their aptitude for rescue in-vitro and in-vivo will translate to success in medical practice has yet to be seen; at the moment, only two colloquially-considered natural products have cleared at least Phase I of clinical testing for CIT therapy: Ginko Biloba Extract, for CIO, and Silymarin, for CIN (Table 3), neither of which predominantly operate through specifically anti-inflammatory mechanisms. While there is a substantial number of naturopathic treatments that test a variety of explicitly anti-inflammatory mechanisms, of which this is not a comprehensive list, there is a distinct lack of an anti-inflammatory focus in the pool of anti-CIT pharmaceuticals. Based on current and extensive reviews on the most promising therapeutics and therapeutic targets, there are really quite few pharmacological intervention options available that are explicitly anti-inflammatory by design. Most of the preclinical and clinical pharmaceuticals under investigation indicate a trend towards a focus on antioxidation, even if several of them do boast potent, secondary, anti-inflammatory qualities.

This is not necessarily unusual, or unexpected. The use of anti-inflammatory drugs can be complicated—especially within the framework of cancer and chemotherapy. The use of anti-inflammatory prescription medication is extremely regulated. Regimens must be well optimised, if not outright personalised, to avoid dangerous levels of immunosuppression and risk of complications. Patients undergoing chemotherapy are often rendered immunosuppressed to some extent already, so the introduction of additional anti-inflammatory agents may certainly prove to be problematic. There is, for instance, quite a complicated relationship between non-steroidal anti-inflammatory drugs (NSAIDs) and cisplatin efficacy already. Most NSAIDs operate by inhibiting the actions of the pro-inflammatory factor, COX2—as many of the preclinical natural and pharmaceutical anti-inflammatory options listed. Unlike those therapeutics, however, meta-analyses have identified cases wherein NSAIDs have, unexpectedly, resulted in either the inhibition of cisplatin treatment or the promotion of CITs—CINs and CICs specifically [311]. Despite this, research into NSAID use during chemotherapy has continued, and several NSAIDs appear capable of improving the outcomes of cisplatin chemotherapy in-vitro and in-vivo [312]. Etoricoxib, as described above, qualifies as a typical NSAID and is representative, in truth, of quite a number of NSAIDs, such as the salicylates, with demonstrable capacity to alleviate CITs, such as CIO and CIN, with little to negative effects on chemotherapy. There are reports of salicylates selectively boosting the cytotoxicity of cisplatin against tumours as chemosensitisers as well [313,314,315,316]. Indeed, there are numerous studies that suggest that anti-inflammatory approaches, even beyond the use of NSAIDs, may actually improve the outcomes of chemotherapy [312,317,318,319,320,321]. More importantly, literature detailing the potential of aforementioned compounds (Table 1 and Table 3) as chemosensitisers for chemotherapy—cisplatin included—is growing likely extensively enough to warrant its own review [322,323,324,325,326,327]. 

As such, there is still a substantial area of research open in CIT therapeutics for targeted, anti-inflammatory therapies that would be designed to interfere with the initiation of the inflammatory signalling cascade, a prospect that is not available for therapies that rely on scavenging of ROS. Given the current landscape of this research and its shift from anti-oxidative to anti-inflammatory therapies, we are closer than ever before to understanding the mechanism of CIT and intercepting the pathways to inflammation, and subsequent ROS generation, to prevent toxicities before they are able to occur. Further insights into these pathways and development of combinatorial therapies with cisplatin and inflammation-based protectants may play an important role in improving long-term health outcomes for cancer patients. 

## Figures and Tables

**Table 1:** Examples of Prospective Natural Anti-Inflammatory CIT Remedies.

Classification	Name	Anti-Inflammatory Mechanism(s) of Action	Targeted CITs	Reference(s)
Non- Flavonoid Polyphenol Phytoalexin	Resveratrol	Inhibits TLR4 Signalling Inhibits IL-6, TNF-α, Inhibits NF-κB Signalling Radical Scavenging Metal Chelation	CIO CIOV (Ovarian) CIU (Uterine) CIN	[165,166,167,168,169,170,171,172,173,174,175,176]
Bee Products	Honey	Inhibits IL6, TNF-α, MCP-1, ICAM1	CIN	[177]
	Caffeic Acid	Radical Scavenging	CIO	[178]
	Bee Pollen Extract	Inhibits IL6, IL1β Inhibits NF-κB Signalling	CIN CIH	[179]
	Mellit (Bee Venom)	Inhibits IL-6, IL-1β, TNF-α Upregulates IL-10 Alters/Inhibits Mθ Invasion Enhanced T-Reg Activity Inhibits COX2	CIN CIPN (Potential)	[180,181,182]
Flavonoid	Curcumin	Inhibits IL-6, IL-1β, TNF-α, MCP-1 Upregulates/maintains IL-10 Free Radical Scavenging Inhibits COX Inhibits JAK/STAT Signalling Inhibits NF-κB Signalling	CIN CIO CIPN CIH	[183,184,185,186,187,188,189,190,191,192,193]
	Epigallocatechin Gallate (EGCG)	Inhibits MAPK Signalling Dis-regulates STAT1/STAT3 Signalling Inhibits IL-6, IL-1β, TNF-α Inhibits NF-κB Signalling Upregulates Nrf2	CIN CIH CICN (Cerebral Neurotoxicity)	[194,195,196,197,198,199]
	Quercetin	Inhibits NF-κB Signalling Potential COX Inhibition Potential iNOS Inhibition	CIO CIN	[200,201,202,203,204,205]
	Baicalein	Inhibits IL-6, TNF-α Inhibits NF-κB Inhibits TLR-2, TLR-4 Signalling Upregulates Nrf2 Upregulates HO-1	CIN	[93,206]
	Wogonin	Inhibits IL-6, IL-8, IL-1β, MCP-1, TNF-α Inhibits NF-κB Signalling Inhibit COX2	CIN	[207,208,209]
	Glycyrrhizic Acid (Licorice)	Inhibits IL-1β, TNF-α Inhibits NF-κB Signalling Reduces DAMP Release	CIN CIH	[210,211,212]
	Hesperetin	Inhibits MAPK Signalling Upregulates TNF-α Signal Inhibitor Upregulates Nrf2 Upregulates HO-1	CIN	[213]
	D-Pinitol	Inhibits IL-6, IL-1β, TNF-α	CIN	[214]
	Sappanone A	Inhibits IL-6, IL-1β, TNF-α Inhibits NF-κB Signalling Inhibits COX2 Upregulates Nrf2	CIN	[215,216,217]
	Xanthohumol	Inhibits IL-6, IL-1β, TNF-α Inhibits TLR4 Expression Inhibits NF-κB Signalling	CIN	[218]
	Puerarin	Inhibits IL-6, TNF-α Inhibits TLR4 Signalling Inhibits ΝF-κB	CIN CIO	[219,220]
	Icariin	Inhibits IL-1β, TNF-α Inhibits NF-κΒ Signalling Inhibits iNOS	CIN	[221,222]
	Genistein	Inhibits ICAM-1, MCP-1 Inhibits Mθ Infiltration Inhibits NF-κB Signalling	CIN CIO	[223,224]
	Galangin	Inhibits IL-6, IL-1β, TNF-α Inhibits MAPK Signalling Inhibits NF-κΒ Signalling	CIN	[225,226]
	Luteolin	Inhibits TNF-α Inhibits NF-κB Signalling Inhibits COX2	CIN	[227]
Saponins	Ginsenosides (Several)	Inhibit IL-1β, TNF-α Inhibit NF-κB Signalling Inhibit COX2 Inhibit iNOS	CIN CIO	[228,229,230,231,232,233]
Alkaloids	Berberine	Inhibits IL-6, IL-1β, TNF-α Inhibits MAPK Signalling Inhibits NF-κB Signalling Upregulates IL-10 s Antioxidant Effect (Reduced ROS Generation)	CIPN CIO	[234,235]
	Betaine	Inhibits IL-6, IL-1β, TNF-α Inhibits NF-κB Signalling	CIN	[236,237]
	Tetramethylpyrazine	Inhibits IL-1β, TNF-α Inhibits TLR4 Signalling Reduces DAMP Release Upregulates Nrf2s	CIN CIO (Potential)	[238,239]
Other Natural Compounds	Astragaloside	Inhibits IL-1β, TNF-α Inhibits Caspase-1 & NLRP3 Inhibits NF-κB Signalling Upregulates Nrf2	CIN	[113,240]
	Sinapic Acid	Inhibits IL-6, IL-1β, TNF-α Inhibits NF-κB Signalling Upregulates Nrf2 Upregulates HO-1	CIN	[241,242]
	Vanillin	Inhibits IL-18, TNF-α Inhibits NF-κB Signalling Inhibits iNOS Upregulates IL-10 Upregulates Nrf2	CIN	[243,244]

**Table 2:** Examples of preclinical anti-inflammatory therapeutics for cisplatin-induced toxicities.

Classification	Name	Anti-Inflammatory Mechanism(s) of Action	Targeted CITs	Reference(s)
Anti-Inflammatory (Iridoid Glycoside)	Aucubin	Inhibits TNF-α Inhibits STAT3/MAPK Signalling Inhibits NF-κB Signalling Upregulates HO-1	CIN	[245]
Anti-Inflammatory (COX2 Inhibitor)	Etoricoxib	Inhibits TNF-α Inhibits iNOS Inhibits COX2	CIN	[173]
Anti-Inflammatory (Clove Oil)	Eugenol	Inhibits IL-6, TNF-α Inhibits COX2	CIO CIN CITT (Testicular)	[246,247,248,249]
Anti-Inflammatory (Iridoid Monoterpinoid)	Monotropein	Upregulated HO-1 Upregulated Nrf2	CIN	[250,251,252]
Anti-Inflammatory (Synthetic Luteolin)	Isoorientin	Inhibits MAPK Signalling Reduces DAMP Release Inhibits NF-κB Signalling Upregulated HO-1 Upregulates Nrf2	CIN	[253]
Anti-Inflammatory (Calcium Antagonist)	Flunarizine	Inhibits IL-6, IL-1β, TNF-α Inhibits MAPK Signalling Inhibits NF-κB Signalling Upregulates Nrf2 Upregulates HO-1	CIO CIUN (Uremic Neuropathy)	[75,254,255,256]
Anti-Inflammatory	R-Phenylisopropyladenosine (R-PIA)	Inhibits TNF-α Inhibits MAPK Signalling Inhibits STAT1 Signalling Inhibits iNOS Inhibits COX2	CIO	[257]
Solubilised Membrane Protein	Thrombomodulin Alfa	Thrombin-Mediated DAMP Degradation	Chemotherapy-IPN	[258,259,260]
Anti-Inflammatory (Nrf2 Activator)	Dimethyl Fumarate	Inhibits Immune Cell Infiltrations Upregulation of Nrf2s Inhibits IL-6, IL4, IL-10, TNF-α	Chemotherapy-IPN CIPNs CIN	[261,262,263]

**Table 3:** Therapeutics for CITs in clinical trials [264,265].

Clinical Trial Status	Intervention (Name)	Mechanism(s) of Action	Listed Target Condition (CIT)	Additional Reference(s)
Recruiting	N-Acetylcysteine	Antioxidant	CIO (CIN)	[266,267,268,269]
Terminated	Sodium Thiosulfate (Trans-Tympanic Gel)	Antioxidant	CIO	[270,271]
Completed	Ginkgo Biloba Extract (GBE761)	Antioxidant Anti-Inflammatory	CIO	[272,273,274,275]
Completed	Sodium Thiosulfate	Antioxidant	CIO	[270,271]
Not Recruiting	Sodium Thiosulfate (+Mannitol)	Antioxidant	CIO	[270,271]
Recruiting	Rosuvastatin	Cholesterol Reduction (HMG-CoA Inhibition) Antioxidant Anti-Inflammatory	CIO CIN	[276,277,278,279,280,281,282]
Completed	Dexamethasone (Synthetic Corticosteroid)	Anti-Inflammatory Antioxidant	CIO	[283,284,285,286,287,288]
Terminated	OTO-104 (Dexamethasone Hydrogel)	Anti-Inflammatory Antioxidant	CIO	[289]
Recruiting	Tempol (SOD and Catalase Mimetic)	Antioxidant	CIN CIO	[290,291,292,293]
Completed	Silymarin	Antioxidant Anti-Inflammatory	CIN	[294,295,296]
Completed	Mannitol (Intravenous)	Osmotic Diuretic	CIN	[297,298,299,300]
Completed	Preloaded Magnesium	Homeostatic Cisplatin Efflux Regulation (Downregulated Transporters)	CIN	[301,302,303,304,305,306]
Completed	Acetazolamide + Mannitol, (+N-Acetylcysteine)	Alkaline Diuretic (Carbonic Anhydrase Inhibitor)	CIN	[307,308]
Recruiting	Pantoprazole	Cisplatin Influx Regulation (OCT2 Inhibitor) Anti-Inflammatory	CIN	[309,310]
